# Mitochondrial Protein UCP1 Inhibits the Malignant Behaviors of Triple-negative Breast Cancer through Activation of Mitophagy and Pyroptosis

**DOI:** 10.7150/ijbs.68438

**Published:** 2022-04-18

**Authors:** Jing Xia, Changbin Chu, Wanqing Li, Hong Chen, Wenhua Xie, Rui Cheng, Kai Hu, Xi Li

**Affiliations:** 1 Institute of Life Sciences, School of Basic Medicine, Chongqing Medical University, Chongqing, 400016, China; 2 Key Laboratory of Diagnostic Medicine Designated by the Chinese Ministry of Education, Chongqing Medical University, Chongqing, 400016, China

**Keywords:** UCP1, Mitophagy, Pyroptosis, GSDME, Triple-negative breast cancer

## Abstract

Triple-negative breast cancer (TNBC) is a massive threat to women's health due to its high morbidity, malignancy, and the refractory, effective therapeutic option of TNBC is still deficient. The mitochondrial protein showed therapeutic potential on breast cancer, whereas the mechanism and downstream pathway of mitochondrial uncoupling protein 1 (UCP1) was not fully elucidated. We found that UCP1 was negatively regulated to the process of TNBC. Overexpressing UCP1 could inhibit the proliferation and metastasis of TNBC, meanwhile inducing the mitochondrial swelling and activation of mitophagy *in vitro*. Mitophagy activation was then assessed to elucidate whether it was downstream of UCP1 in TNBC metastasis. GSDME is the core of pyroptosis. We found that GSDME was activated in the TNBC cells when UCP1 levels were high. It regulates TNBC cell proliferation potential instead of the apoptosis process *in vitro* and *in vivo*. Our results suggested that UCP1 could inhibit the process of TNBC by activating mitophagy and pyroptosis. Impaired activation of mitophagy weakens the regulation effect of UCP1 on metastasis of TNBC, similar to the impairment of GSDME activation on the proliferation regulation of UCP1 on TNBC. UCP1 might be a novel therapeutic target of TNBC.

## Introduction

Breast cancer is the highest incidence cancer among adult women 1 due to its heterogeneity and drug resistance. The exact mechanism of the development of breast cancer remains scientifically unclear.

Breast tissue is mainly composed of adipocytes surrounding mammary glands physiologically. Lipids and their metabolism are essential for naturally maintaining breast tissue function. When malignancies appear in breast tissue, lipid metabolism is reprogrammed synchronously.

Lipid metabolism accelerates the process of breast cancer through energy support and membrane construction^2^ and modulates the drug resistance and prognosis on various subtypes of breast cancer^3^. These studies revealed lipid metabolism's importance and therapeutic potential on breast cancer.

Mitochondrial homeostasis is crucial for the cellular process. As a core organelle of lipid metabolism, mitochondria are fully recognized as a 'powerhouse'. Targeting mitochondrial metabolism therapy was effective even in triple-negative breast cancer 4, 5, a most heterogeneous, malignant, and drug-less subtype of breast cancer. It showed the target therapeutic potential of mitochondrial on breast cancer, and the further mechanism of mitochondrial regulation on breast cancer was still deficient.

Mitochondrial uncoupling protein 1(UCP1), a member of the family of mitochondrial anion carrier proteins (MACP), separates the H^+^ stream from ATP/NADPH synthesis. UCP1 can regulate the membrane potential balance and also show long-chain fatty acid-binding potential, which helps maintain the lipid metabolism balance^6^. In non-small cell lung cancer, UCP1 was highly expressed with increased glucose absorption^7^. However, UCP1 was down-regulated in colorectal cancer while positively related to a better prognosis 8. As a core factor of lipid catabolism and non-shivering thermogenesis, UCP1 'slimmed' clear cell renal cell carcinoma, leading to lipid browning and impairing tumor progress^9^, ^10^. Those inhibition effects were similar in the breast cancer cell line HCC1806^11^. In ALDH-positive breast cancer stem cells, UCP1 could reduce the inhibitory effect of Snail on glucose metabolism enzyme FBP1, suppressing the tumor progress^12^. Those researches showed a therapeutic potential of UCP1 on cancer through glucose and lipid metabolism regulation, whereas the mitochondrial regulation potential of UCP1 on breast cancer was insufficient.

Overexpressed UCP1 induces H^+^ efflux of the mitochondrial membrane. A high level of H^+^ efflux decreases mitochondrial membrane potential, resulting in mitochondrial dysfunction and thus inducing mitophagy^13^,^14^. The effect of mitophagy on the breast cancer process was inconclusive. Studies showed that mitophagy might partly respond to doxorubicin resistance^15^ and inhibit warangalone-induced mitochondrial apoptosis in breast cancer cells^16^. Those studies showed that breast cancer regulates the potential of mitophagy; UCP1 may induce mitochondrial self-repair program, yet with uncertain effect and mechanism. Mitophagy was also found to inhibit the maintenance of breast stem cells 17 and was the core process of PARP inhibitor-induced cell death 18. However, some other downstream cascade reactions, like apoptosis and pyroptosis, initiated secondary to UCP1 induced mitochondrial dysfunction, were still unclear. Other effects and mechanisms on the regulation of UCP1 on breast cancer, especially in triple-negative breast cancer, were still urgently needed.

The present study showed that UCP1 regulates the proliferation and metastasis of triple-negative breast cancer (TNBC) *in vitro* and *in vivo* by activating mitophagy and pyroptosis. Our data stressed the importance of UCP1 on the development of TNBC and provided a potential therapeutic target of TNBC.

## Materials and methods

### Bioinformatics analysis

Data downloaded from The Cancer Genome Atlas (TCGA) was analyzed through *ualcan.path.uab.edu* and* GEPIA*. Data downloaded from Kyoto Encyclopedia of Genes and Genomes (KEGG) was analyzed through Gene Ontology (Go) analysis, cut-off value: fold change>2 and p<0.05.

### Immunohistochemical (IHC) staining

The breast tissue microarray was purchased from Ailina biotechnology (Xi'an, China). The ectopic transplanted tumor of the nude mouse was fixed in 4% paraformaldehyde, embedded in paraffin and cut into slides with a thickness of 5 μm. The microarray and slides were subjected to antigen retrieval before incubating with the appropriate antibodies. Expression of UCP1 (Abcam, Ab155117), GSDME (Abcam, Ab230482) and Ki67 (Abcam, Ab15580) were detected.

### Cell culture

Triple-negative breast cancer cell line MDA-MB-231 was cultured with 10%fetal bovine serum (Gibco, United States) in 4.5g/L glucose Dulbecco's modified Eagle medium (DMEM) (Gibco, United States) with 1% penicillin/streptomycin (p/s) (Beyotime, China). Triple-negative breast cancer cell line BT549 was cultured with 10%fetal bovine serum (Gibco, United States) in Roswell Park Memorial Institute (RPMI) 1640 with 1% p/s. Breast endothelial cell line MCF-10A was cultured in MCF-10A special medium (Procell, China). All the cells were cultured at 5% CO_2_, 37℃.

### Western Blot

Total protein was extracted using cell lysis buffer (with protease and phosphatase inhibitor), and protein concentration was determined using Nanophotometer (Implen, Germany). The proteins were separated with 5%, 10% or 15%SDS-polyacrylamide gel electrophoresis (SDS-PAGE) (based on protein molecular weight), and transferred to polyvinylidene difluoride (PVDF) membranes (Merck Millipore, United States). The membranes were blocked at 5% skim milk for 1.5h at room temperature, and then blotted with primary antibodies at 4°C overnight. The secondary antibody (Jakson, United States) was diluted at 1:10000 in 5% skim milk and treated the membrane for 1h at room temperature. Pico plus ECL reagent solution kit (Share Bio, China) and Amersham Imager 600 (General Electric Company, United States) were used to detect the immunoreactive bands. Table 1 shows the list of antibodies used for Western Blot.

### Reverse transcription (RT) and quantitative real-time PCR (qPCR) analysis

TRIzol™ reagent (Invitrogen, United States) was used to isolate total RNA. Complementary DNA (cDNA) was synthesized through RevertAid First Strand cDNA Synthesis kit (Thermo scientific, United States) by Two-Step cDNA Removal according to the manufacturer's instructions. qPCR was progressed by PowerUp™ SYBR™Green Master Mix (Thermo scientific, United States) in Quantstudio3/5 (Thermo Scientific, United States) real-time PCR instrument. The expression levels of target genes were calculated using the 2-ΔΔCT method with normalization to the standard housekeeping gene 18s, and expressed as relative mRNA levels compared with internal control. Those primers were used: UCP1 (Forward: 5'- CAATCACCGCTGTGGTAAAAAC-3'; Reverse: 5'-GTAGAGGCCGATCCTGAGAGA-3'); beta-actin (Forward 5'-CATGTACGTTGCTATCCAGGC-3'; Reverse: 5'-CTCCTTAATGTCACGCACGAT-3').

### Lentivirus construction

PCDH-CMV-MCS-EF1-puro and PCDH-CMV-MCS-EF1-GFP-puro were purchased from Hanbio Biotechnology (Shanghai, China). Lentivirus of UCP1 overexpression used following primers: sense 5'- CTAGCTAGCTAGATGGGGGGCCTGACAGCCTCGGACG-3'; antisense 5'- AACTGCAGAACCAATGCATTGG CGTCCGAGGCTGTCAGGCCCCCCAT-3'. The UCP1 overexpression and vector lentivirus were constructed according to the manufacturer's protocol, and were transfected using Lipofectamine 3000 (Thermo, United States) according to the manufacturer's protocol.

### Cell proliferation Determination

To measure the proliferation potential of TNBC cells, Cell Counting Kit-8 (CCK8, Dojindo Laboratories, Japan) was used. MDA-MB-231 and BT549 with vector/UCP1 overexpressed were seeded into 96 well with 3×10^3^/well cell culture clusters, and other specific operation steps were followed according to the manufacturer's protocol.

### Flow Cytometry Analysis

Flow Cytometry analysis was used to measure the cell cycle, apoptosis and membrane potential. To detect the cell cycle change, MDA-MB-231 and BT549 with vector or UCP1 overexpressed were isolated and fixed by 70% ethanol, at 4℃, overnight. The next day, cells were centrifuged by 1000rpm for 3min, the supernatant was discarded, and the cells were resuspended and stained by diluted propidium (PI) (Beyotime, China). The cell cycle was measured by BD Accuri C6 Plus (BD, United States), and analyzed with Flow Jo V10.

To detect the apoptosis change of TNBC cells, MDA-MB-231 and BT549 with vector/UCP1 overexpressed were resuspended with PBS and centrifuged by 1000rpm for 3min, discard the supernatant. The cells were then resuspended and stained by Annexin V-PE staining (Beyotime, China) as suggested by company. The cell apoptosis was measured by BD Accuri C6 Plus (BD, United States).

To measure the membrane potential change of TNBC cells, MDA-MB-231 and BT549 with vector/UCP1 overexpressed were resuspended with PBS and centrifuged by 1000rpm for 3min, discard the supernatant. The cells then were resuspended and stained by Image-iT™ TMRM (Invitrogen, United States) for 30min, 37℃. The result was measured BD Accuri C6 Plus (BD, United States).

To measure the Reactive Oxygen Species (ROS) change of TNBC cells, MDA-MB-231 with vector/UCP1 overexpressed were resuspended with PBS and centrifuged by 1000rpm for 3min, then discard the surpernatant, resuspend and stained by MitoSOX™Red mitochondrial superoxide indicator (Invitrogen, United States). The specific operation steps were followed as manufacturer's protocol suggested.

### Transwell assay

Transwell chambers (8μm polycarbonate filter, Corning, United States) were used to evaluate cell metastasis and invasion potential.MDA-MB-231 (5×10^4^) in DMEM without serum and BT549 (5× 10^4^) in RPMI 1640 medium without serum were seeded into the upper chamber without/with diluted (1:200 in medium) matrigel matrix (Corning, United States). The lower chamber was filled with 700 μl medium with 10% FBS as a chemoattractant. After 12h of incubation, the chambers were isolated, and the cells on the upper surface of the transwell membrane were removed. Cells on the lower surface were fixed into 4% paraformaldehyde, 15min, and stained with 0.01% crystal violet solution (Beyotime, China), 10 min.

### Xenograft Studies

10 four-week-old female BAL/c nude mice (Charles River, China) were sacrificed in this study. UCP1 overexpressed or vector control MDA-MB-231 cells (1 × 10^7^) were mixed with 200μl of phosphate-buffered saline (PBS),4℃. The cells were subcutaneously inoculated into the right axilla of mice. The diameter of the tumor was measured every 3 days with a caliper. 21 days after inoculation, the mice were euthanized. The tumor volume was detected by converting into equal volume of water, and the weight and tumor of mice were detected by precision balance. The mice experiments were performed in accordance with the National Institutes of Health Guide for the Care and Use of Laboratory Animals with approval from the Ethics Committee of Chongqing Medical University.

### *In vivo* metastasis study

12 four-week-old female BAL/c nude mice (Charles River, China) were sacrificed in this study. UCP1-GFP overexpressed or vector-GFP control MDA-MB-231 cells (2 × 10^6^) were mixed with 200μl of phosphate-buffered saline (PBS), at 4℃. The cells were inoculated through the tail vein into mice in 40-60s to avoid embolism formation. Weight change was measured every 3 days, the mice were euthanized 35 days after inoculation. Lung, liver and kidney of mice were detected. The mice experiments were performed in accordance with the National Institutes of Health Guide for the Care and Use of Laboratory Animals with approval from the Ethics Committee of Chongqing Medical University.

### Immunofluorescence (IF) staining

Immunofluorescence was performed to evaluate the location of UCP1 in mitochondrial.MDA-MB-231 cells with stable UCP1 overexpressed were cultured in 6 well cell culture clusters with slides to get cell crawling slides. The slides was fixed in 4% paraformaldehyde, 30min, and then treated with 0.5%Triton X-100 in room temperature, 30min. 5% normal donkey serum was used to block sides of slide, 2h in room temperature and added UCP1 antibody (PBST dilution: 0.1% Tween-20 and 0.5% BSA, 1:100 dilution) dropwise and incubated the slides in a wet box at 4°C overnight. The next day, rewarming the slides for more than 30 min, adding corresponding fluorescent secondary antibody (1:500 dilution) and incubating it at room temperature for 1h. Cell nuclear was stained with DAPI (4',6-Diamidino-2-28 phenylindole dihydrochloride) for 10 min at room temperature, and mitochondrial was stained by Mito-Tracker Red CMXRos (Beyotime, China). The fluorescence was detected by a confocal microscope (Olympus, Japan).

### Transmission electron microscope detection

MDA-MB-231 with vector or UCP1 overexpressed was collected, 800rpm, 5min, fixed in a special fixator and stored at 4℃. The structure of mitochondria was detected by JEOL JEM-1400 PLUS transmission electron microscope (Nippon Electronics Co, Japan).

### Release of Lactic dehydrogenase (LDH) Determination

To measure the release of LDH on TNBC cells, LDH Cytotoxicity ƒAssay Kit (Beyotime,China) was used. MDA-MB-231 and BT549 with vector/UCP1 overexpressed were seeded into 96 well with 3×10^3^/well cell culture clusters, and other specific operation steps were followed according to the manufacturer's protocol.

### Cell treatment of inhibitors

MDA-MB-231 and BT549 with vector or UCP1 overexpressed was cultured in 5% CO_2_, 37℃ and was treated with vehicle, 5μM or 10Μm Cyclosporine A (CsA, selleck, S2286,China) for 24h. MDA-MB-231 and BT549 with vector or UCP1 overexpressed was cultured in 5% CO_2_,37℃ and was treated with vehicle or 60μM Z-DEVD-Fmk(selleck, S7312,China) for 75min, then remove the medium and added fresh culture medium(described in 'cell culture'), 5% CO_2_, 37℃,24h,respectively.

### Statistical Analysis

We performed all experiments in at least triplicate. All data are presented as means ±standard errors of the mean (SEM). Mean values (continuous variables) between two groups were assessed by two-tailed Student's t-test and two-way ANOVA. P values<0.05 were considered statistically significant. Statistical analyses were performed with Graph Pad Prism 5 (GraphPad Software Inc., USA).

## Results

### UCP1 is positively related to a better prognosis of breast cancer

**Figure 1:**
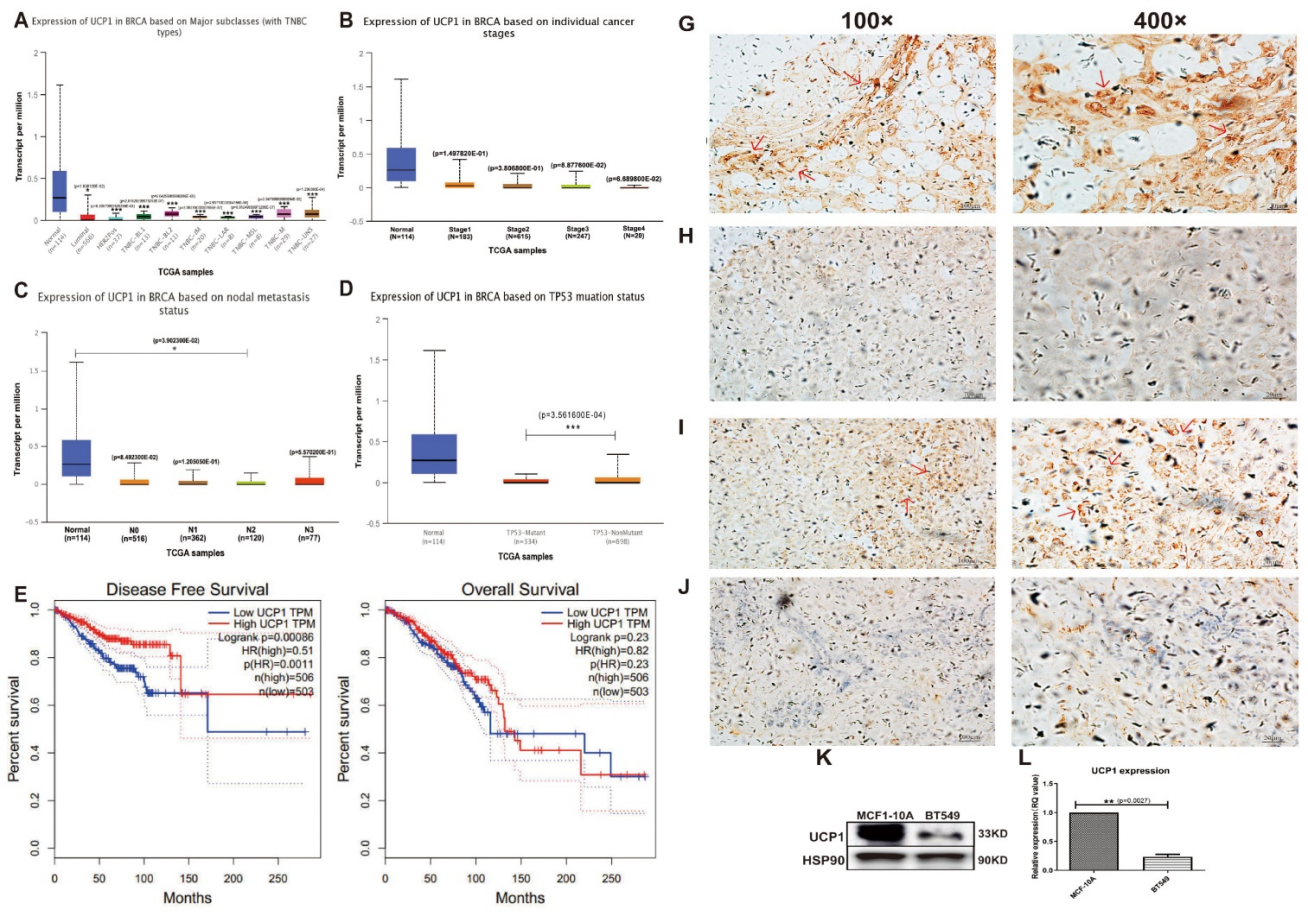
** UCP1 was positively related to a better prognosis of breast cancer. (A)** Expression of UCP1 in normal breast tissue (n=114), luminal (n=566), HER2 positive (n=37) and different triplenegative (n=153) subclass. **(B)** Expression of UCP1 based on breast cancer individual stages. Normal (n=114), stage 1 (n=183), stage 2 (n=615), stage 3 (n=247), stage 4 (n=20). **(C)** Expression of UCP1 based on nodal metastasis. Normal (n=114), N0 (n=516), N1 (n=362), N2 (n=120), N3 (n=77). **(D)** Expression of UCP1 based on TP53 mutation status. TP53 mutant (n=334), TP53 non-mutant (n=698) **(E)** Disease free survival curve of high UCP1 expression and low UCP1 expression, present by months. **(F)** Overall survival curve of high UCP1 expression and low UCP1 expression, present by months. **(G)** Immunohistochemical staining of UCP1 in adenosis sample (normal breast control), **(H)** invasive ductal carcinoma, **(I)** neuroendocrine carcinoma, and **(J)** invasive lobular carcinoma. **(K)** Protein and **(L)** mRNA expression of UCP1 in MCF-10A and BT549.*:p<0.05,**:p<0.01,***:p<0.001

Data collected from TCGA showed expression of UCP1 decreased significantly on major breast cancer subclasses, like HER2+ and triple-negative, rather than on normal breast tissue (Fig. 1A). Individual cancer stage and node metastasis status showed a similar positive relationship between the expression of UCP1 and better prognosis (Fig. 1B, 1C). TP53 mutation is an admitted tumor-promote biomarker around cancers, the expression of UCP1 on TB53-mutant status was decreased, compared to TP53-nonmutant status (Fig. 1D). For more than a decade (130 months), comparing the breast cancer patients with low UCP1 expression, high UCP1 expression individuals have a higher rate of disease-free survival and overall survival (Fig. 1E, 1F).

Immunohistochemical staining detected a significant expression of UCP1, in the adenosis sample (normal breast control), on the mammary gland tube (Fig. 1G, red arrow, 100X and 400X), and a rare expression in invasive ductal carcinoma (Fig. 1H, 100X and 400X), a malignant subtype breast cancer with poor prognosis. Expression of UCP1 was also found in neuroendocrine carcinoma (Fig 1I, red arrow, 100X and 400X), and rare expression in invasive lobular carcinoma (Fig 1J, 100X and 400X), another malignant subtype of breast cancer.

Furthermore, we detected the UCP1 expression of protein and mRNA on MCF10A (an immortalized standard breast cell line) and in BT549 (a triple-negative breast cancer cell line). Compared with MCF10A cells, the expression of UPC1 was remarkably decreased on both protein and mRNA levels in the BT549 cell line (Fig. 1K, 1L).

These data showed that UCP1 was down-regulated on both breast cancer tissue and breast cancer cell lines and positively related to a better breast cancer prognosis. It suggested a potential effect of UCP1 on the repression of breast cancer.

### Overexpression of UCP1 blunts the malignancy of triple-negative breast cancer *in vitro* and* in vivo*

**Figure 2:**
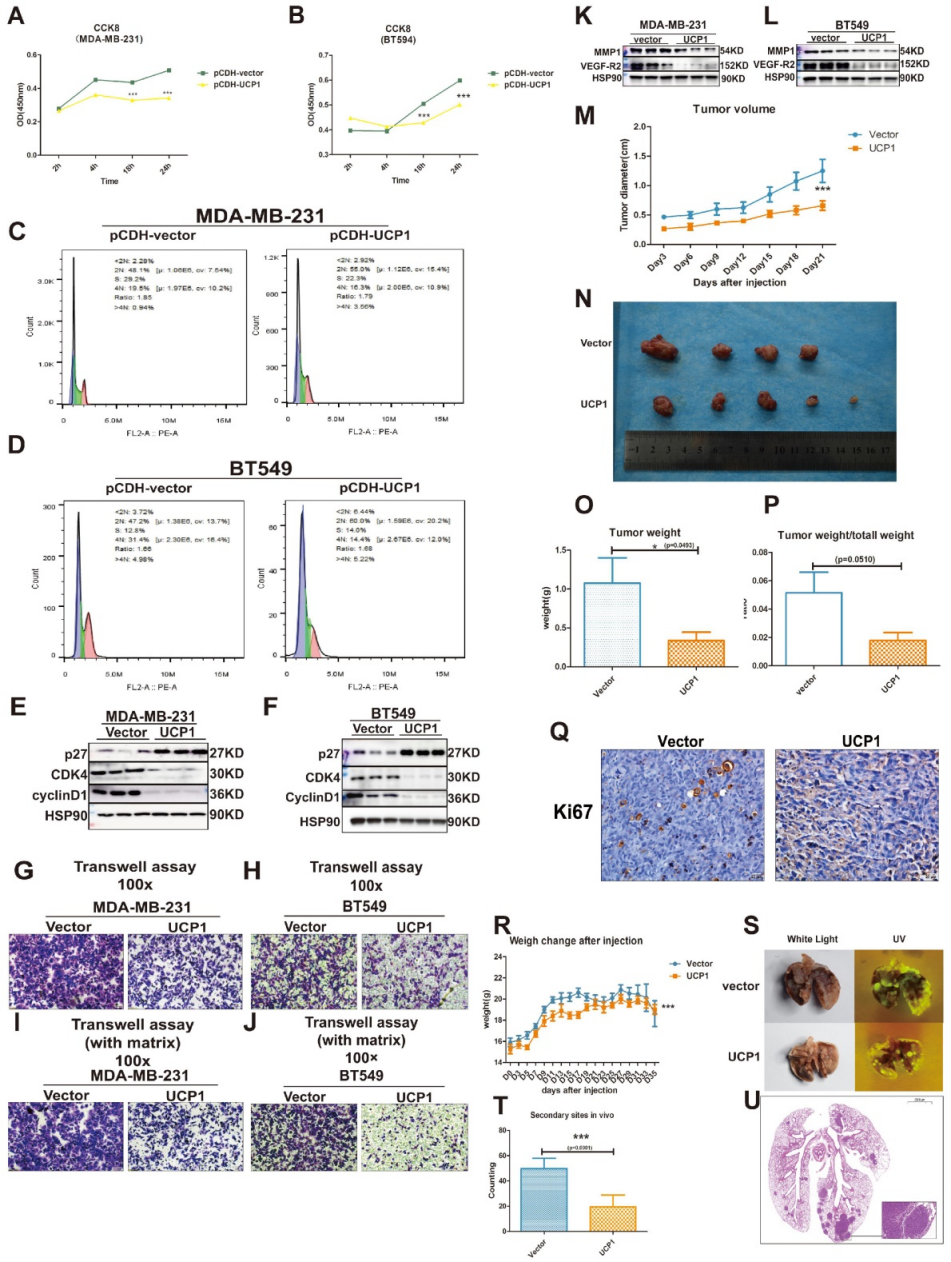
** Overexpression of UCP1 could impair the malignant behavior of triple-negative breast cancer. (A)** CCK8 assay of MDA-MB-231 and **(B)** BT549 with vector or UCP1-overexpressed. **(C)** Cell cycle analysis of MDA-MB-231 and **(D)** BT549 with vector or UCP1-overexpressed. **(E)** Protein expression of cell cycle makers of MDA-MB-231 and **(F)** BT549 with vector or UCP1-overexpressed. **(G)** Transwell assay of MDA-MB-231 and** (H)** BT549 with vector or UCP1-overexpressed. **(I)** Transwell assay with matrigel of MDA-MB-231 and** (J)** BT549 with vector or UCP1-overexpressed. **(K)** Protein expression of metastasis factors in MDA-MB-231 and** (L)** BT549 with vector or UCP1-overexpressed. **(M)** Diameter of subcutaneous transplantation tumor by days. **(N)** Subcutaneous transplantation tumor of MDA-MB-231 with vector (upside) and UCP1 overexpressed (downside). **(O)** Tumor weight and **(P)** Tumor weight compared with total mouse weight of subcutaneous transplantation tumor with vector or UCP1 overexpressed. **(Q)** Ki67 stain of subcutaneous transplantation tumor with vector or UCP1 overexpressed. **(R)** Mouse body weight change after triple negative breast cancer cell line tail vein injection. **(S)** Lung of nude mouse with tail vein injection. Left, white light; right, ultraviolet light (UV). **(T)** The number of secondary sites on mouse lung with tail vein injection. (The counting was standardized). **(U)** Hematoxylin and eosin (he) staining of the lung with secondary sites. Mean+SEM of CCK8 was analyzed by two-tailed Student's t-test, tumor volume and body weight change were analyzed by two-way ANOVA.*: p<0.05, **: p<0.01, ***: p<0.001

Two types of triple-negative breast cancer cell lines, MDA-MB-231 and BT549, were chosen as target cell lines to investigate whether the overexpression of UCP1 could inhibit the malignant behavior of breast cancer. Compared with the MDA-MB-231 and BT549 cells transfected with the control vector, proliferation potential was significantly decreased after 18h and 24h in UCP1 overexpressed group (Fig. 2A, 2B). Enrichment analysis shows that the cell cycle may be a downstream target of UCP1 (Fig S1). It indicates that overexpressing UCP1 in MDA-MB-231 and BT549 cells induce an increase in the cell number in the G1/S phase and markedly decreases in the G2/M phase (Fig. 2C and 2D). These results were due to the enhanced expression of p27, a negative regulator of cell cycle transition, and reduced cyclinD1 and CDK4 levels (Fig. 2E and 2F), which are classical protein-complex promoting G2/M transition, after UCP1 overexpression. Thus the p27-cyclinD1-CDK4 regulatory axis-activated in MDA-MB-231 and BT549, resulting in cell cycle arrest.

Simultaneously, lymph node metastasis promotes factor, vascular endothelial growth factor (VEGF) and matrix metalloproteinase 1 (MMP1), one key enzyme activating extracellular matrix proteolysis during tumor metastasis, have been down-regulated while overexpressing UCP1 (Fig. 2K, 2L). Vertical migration and extracellular matrix digest capacities of MDA-MB-231 and BT549 with UCP1 stable transfected were reduced (Fig. 2G-J).

*In vivo* analysis, the UCP1 stable-transfected cell line had smaller orthotopic xenograft models in BALB/c nude mouse (Fig. 2M-Q). The cell proliferation marker Ki67 was diminished compared with the control vector-transfected group in triple-negative breast cancer cells (Fig. 2P). Moreover, MDA-MB-231 transfected with UCP1 also showed less quantity and smaller volume of secondary sites (Fig. 2R-U), demonstrating the regulatory potential of UCP1 on metastasis of triple-negative breast cancer *in vivo*. Those data evinced that UCP1 can lessen malignant behaviors on triple-negative breast cancer, both *in vitro* and *in vivo*.

### UCP1 regulates metastasis and proliferation of breast cancer by activating mitophagy and pyroptosis

**Figure 3:**
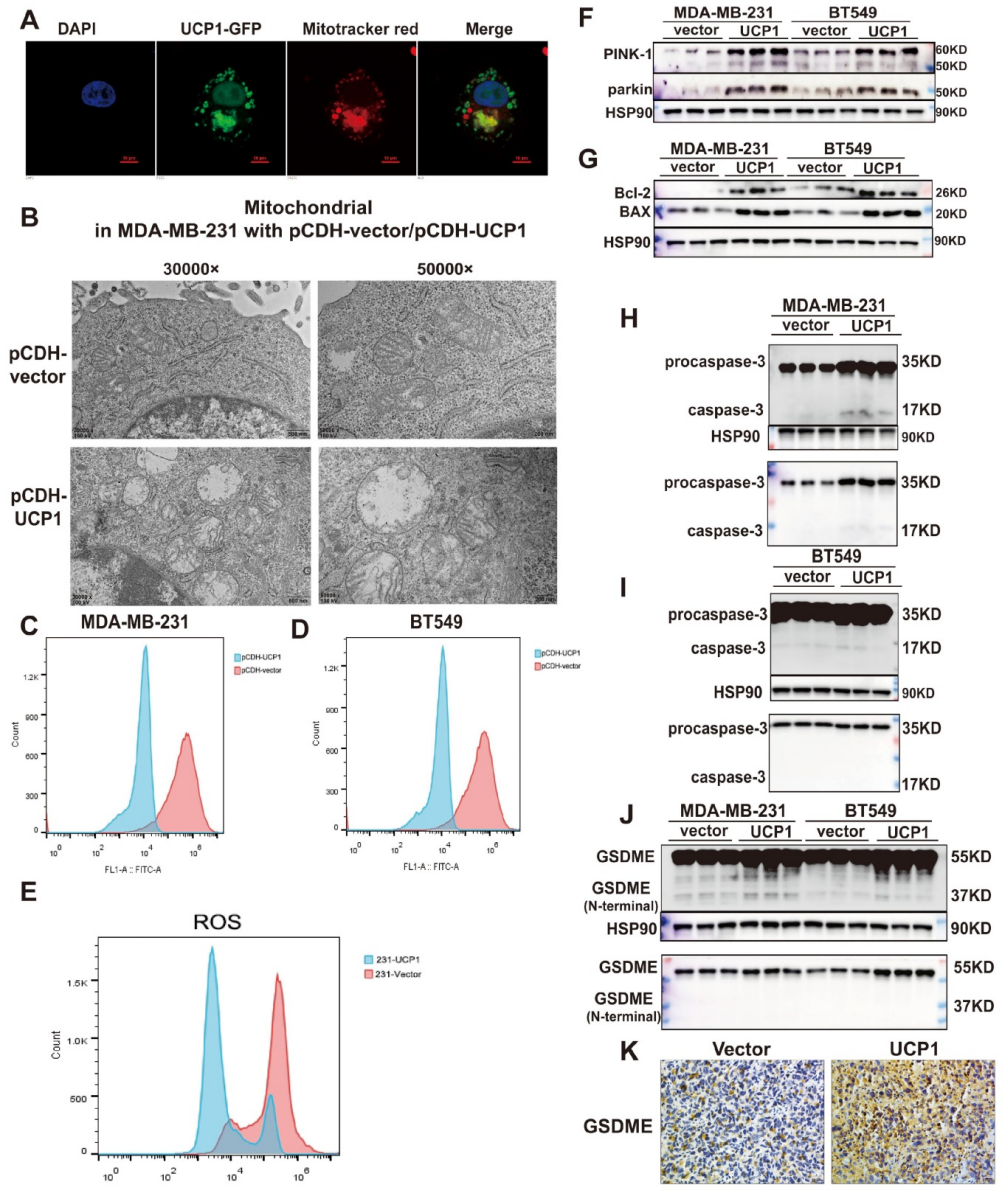
The regulatory effect of UCP1 on the metastasis and proliferation process of breast cancer may through mitophagy and pyroptosis activation. **(A)** Confocal images of UCP1 (green fluorescence) and mitochondrial (red fluorescence) in MDA-MB-231 with UCP1 overexpressed. **(B)** Electron microscope image of MDA-MB-231 with vector or UCP1. **(C)** TMRM stain to detect the membrane potential of MDA-MB-231 and **(D)** BT549 with vector or UCP1 overexpressed. **(E)** Ros level in MDA-MB-231 with vector or UCP1. **(F)** Protein expression of PINK-1 and parkin in MDA-MB-231 and BT549 with vector or UCP1 overexpressed. **(G)** Protein expression of mitochondrial apoptosis factors in MDA-MB-231 and BT549 with vector or UCP1 overexpressed. **(H)** Protein expression of pro-caspase-3 and caspase-3 in MDA-MB-231 and **(I)** in BT549 with vector or UCP1 overexpressed. (J) Protein expression and Immunohistochemical staining **(K)** of GSDME in MDA-MB-231 and BT549 with vector or UCP1 overexpressed.

Proteins exert different functions depending on their subcellular localization. In order to investigate the potential downstream target of UCP1 on the process of breast cancer, we detected whether the overexpressed UCP1 protein is located in mitochondria to exert its function in breast cancer. Confocal images showed that UCP1 (green fluorescence) was co-localized with mitochondria (red fluorescence) in the UCP1 stable overexpressed breast cell line (Fig. 3A). The electron microscope images demonstrated that the mitochondria were swelling, its' cristae structures were disappeared, which tends to a mitochondrial structure destroyed phenotype in UCP1 stable overexpressed breast cancer cell line (Fig. 3B). This result is consistent with the phenotype of UCP1 overexpressed *in situ*. Mitochondrial membrane potential (Figure 3C and 3D) and ROS level (Fig. 3E) were also decreased in TNBC cell lines, confirming the damaged mitochondrial function and the activation of mitophagy. PTEN induced putative kinase-1 (PINK-1), a mitochondrial serine/threonine kinase, is correlated with mitochondrial function. PINK-1 helps parkin translocated to mitochondrial and promotes mitophagy^.[19]^. We observed that the overexpressed UCP1 increased both PINK-1 and parkin expression (Fig. 3F), further proving the activation of mitophagy. We hypothesized that mitochondrial apoptosis was also triggered after UCP1 overexpressed, considering the mitochondrial structure disruption. Therefore, we tested the apoptosis-involved protein expression: the anti-apoptosis protein Bcl-2 and the Bcl-2 associated protein X (BAX). BAX can bind to Bcl-2 and exert an antagonism effect. Our data indicated that both expressions increased after UCP1 overexpression (Fig. 3G). Release of cytotoxic LDH was augmented, whereas apoptosis marker annexin-V was down-regulated after UCP1 overexpression (Fig. S4) in MDA-MB-231cells. These data advised that a non-apoptosis cell death pathway may be active in breast cancer cells with UCP1 treatment.

Pyroptosis is a GSDME mediated and inflammation-related death pathway that differs from apoptosis. Gasdermin E (GSDME), also named DNFA5, could be cleaved by caspase-3, and its' N-terminal mediates the process of secondary cell death activated by BAX 20. Expression of the active form of caspase-3 and GSDME's N-terminal were increased, demonstrating that pyroptosis has occurred or enhanced in MDA-MB-231 and BT549 with UCP1 overexpression (Fig. 3H-J).

### The inhibition of mitophagy and pyroptosis injures the positive effect of UCP1 on TNBC

**Figure 4:**
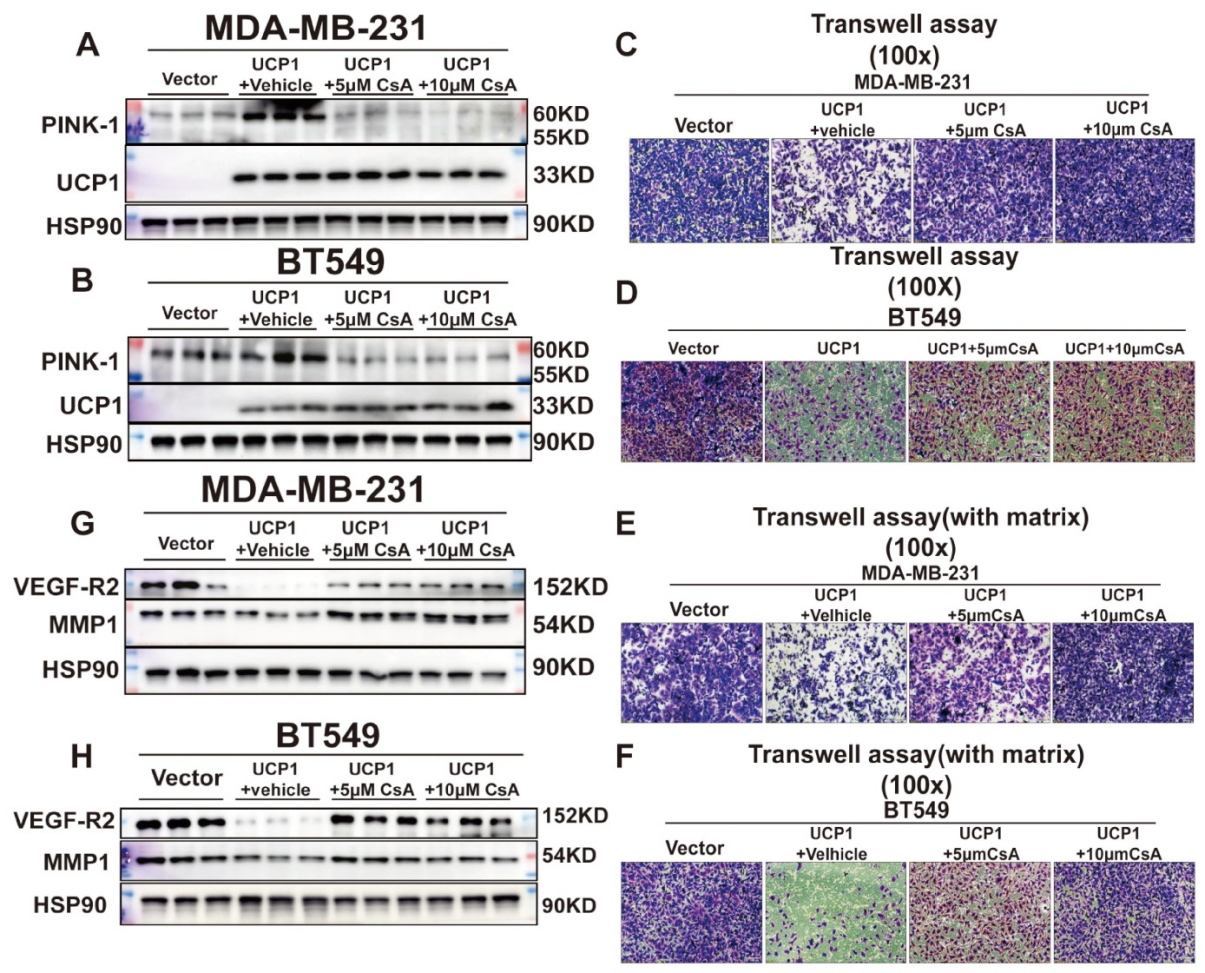
** Mitophagy inhibition could impair the regulatory effect of UCP1 on metastasis and invasion of TNBC. (A)** Expression of PINK-1 in MDA-MB-231and **(B)** BT549 with UCP1 overexpressed after 5μM and 10μM CsA treated. **(C)** Transwell assay of MDA-MB-231 and **(D)** BT549 in groups same as **(A)** and **(B)**. **(E)** Transwell assay (with matrix) of MDA-MB-231 and **(F)** BT549 in groups as indicated. **(G)** Protein expression of regulated factors of invasion on MDA-MB-231 and **(H)** BT549 in groups as indicated.

Mitophagy was essential to maintain mitochondrial health and dynamics. It also showed an inhibitory effect on mammary tumor metastasis^21^,^22^,^23^. To investigate whether UCP1-mediated mitophagy is involved in tumor migration and invasion, cyclosporine A (CsA) was employed to block mitophagy (Fig. 4A, 4B). Transwell assay revealed that the CsA treatment impaired the positive effect of UCP1 on migration and invasion in breast cancer cells (Fig. 4C-F). Furthermore, it inversed the expression level of invasion regulators MMP1 and VEGF-R2 (Fig 4G, 4H) in both TNBC cell lines. These data confirmed that mitophagy was involved in the regulation of UCP1 on migration and invasion in TNBC.

### Inactivation of caspase-3 and GSDME reversed the regulatory effect of UCP1 on the proliferation of TNBC

**Figure 5:**
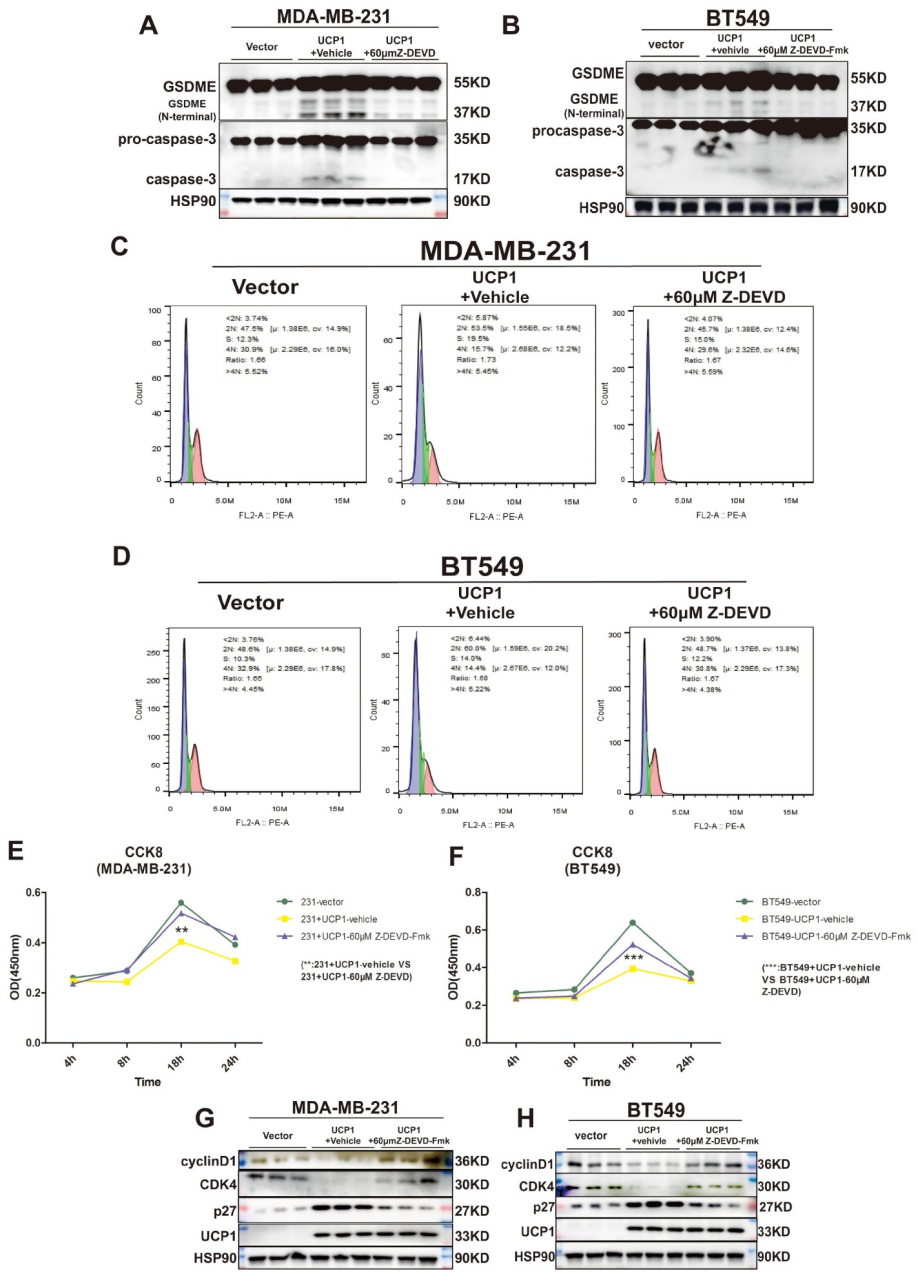
** Inactivation of caspase-3 and GSDME reversed the regulatory effect of UCP1 on the proliferation of TNBC. (A)** Expression of GSDME and caspase-3 with active subunits in MDA-MB-231 and **(B)** BT549 with UCP1 stable transfected, meanwhile after 60μM Z-DEVD-Fmk treated. **(C)** The cell cycle of MDA-MB-231 and **(D)** BT549 in groups same as **(A)** and **(B)**. **(E)** CCK8 assay of MDA-MB-231 and **(F)** BT549 in groups as indicated. **(G)** Western blot of regulated factors of cell cycle and proliferation on MDA-MB-231 and **(H)** BT549 with UCP1 stable transfected after 60μM Z-DEVD-Fmk treatment. **: p<0.01, ***: p<0.001.

GSDME was found to suppress tumor growth by activating pyroptosis and secondary inflammation process 24. To confirm whether pyroptosis could be the intermediary between UCP1 and the proliferation process of TNBC, we inhibited the activity of caspase-3, the upstream of GSDME, and directly cleaved GSDME in MDA-MB-231 and BT549 cells (Fig. 5A, 5B). The negative effect of UCP1 in the cell cycle was weakened (Fig. 5C, 5D), and the cell proliferation of TNBC with overexpressed UCP1 was also rescued after 60μM caspase-3 inhibitor Z-DEVD-FMK treatment (Fig. 5E, 5F). Expression levels of p27, CDK4, and cyclinD1 were reversed (Fig. 5G, 5H), demonstrating that the regulator of proliferation may be the target of GSDME after overexpressing UCP1. Those data evinced that pyroptosis inhibition impairs the regulatory effect of UCP1 on TNBC.

## Discussion

As a member of the family of mitochondrial anion carrier proteins (MACP), UCP1 was mainly considered to separate the H^+^ stream from ATP/NADPH synthesis and lead to the energy dissipated into heat. UCP1 was also found to have the long-chain fatty acid-binding ability 6. Meanwhile, an anti-obesity effect 25, 26 shows its regulatory potential in lipid metabolism and breast cancer. Moreover, UCP1 was reported to regulate the stemness of breast cancer stem cells through glycometabolism key enzyme FBP1 and ALDH 12, revealing various functions of UCP1 in the breast cancer process. The present study demonstrated that overexpressing UCP1 inhibits the endless proliferation potential, and prevents metastasis and invasion capacity by activating mitophagy in TNBC. Simultaneously, overexpressed UCP1 increases the expression of the N-terminal of GSDME. This reinforcing effect on GSDME-mediated pyroptosis by UCP1 has not been reported previously, therefore implying a comprehensive and novel inhibitory potential of UCP1 on TNBC.

The effect of mitophagy activation in cancer was no consensus. Defected mitophagy promotes the proliferation and metastasis of breast cancer *in vivo* 21. Moreover, the loss function of BRCA1 (a major breast cancer suppressor) impairs mitophagy leading to breast cancer metastasis 22. Targeting mitochondrial iron metabolism induced mitochondrial dysfunction and mitophagy, suppressing tumor growth and metastasis 23. Those researches showed the tumor-suppress effect of mitophagy. Nevertheless other studies revealed the reverse tendency of mitophagy on breast cancer progression. PINK/parkin-induced mitophagy was found to inhibit apoptosis 14, and immature mitophagy may be related to breast cancer cell death 27, implying the tumor-promote effect of mitophagy on breast cancer. Our data showed that compared with mitochondrial destruction and dysfunction, the activating function of UCP1 in mitophagy was crucial, thus regulating metastasis and decreasing ROS in TNBC. Increased ROS could drive hypoxia-inducible factor (HIF) stabilization^28^ and inactive of p53-brain angiogenesis inhibitor 1 (BAI1) pathway 29 to maintain the metastasis potential of breast cancer, showing that UCP1 may target ROS to regulate metastasis of TNBC.

ROS was also apoptosis promote factor in breast cancer 30, 31. Interestingly, mitophagy inactivation leading to a proliferation inhibitory effect on TNBC with UCP1 overexpressed (Fig. S2), This partly confirmed the inhibitory effect of mitophagy on apoptosis. Nonetheless, overexpression of UCP1 still exerts an inhibitory effect on TNBC cell proliferation, suggesting a more robust regulatory mechanism existed for UCP1 on the proliferation of TNBC.

Pyroptosis is a non-apoptosis inflammation-related cell death process. It can be initiated by activating inflammasome or other pro-inflammation processes executed by GSDMs and can lead to the secondary inflammatory response in physiological condition. Research showed that pyroptosis and GSDMs have contradictory effects on tumor progress in mammary carcinoma. GSDMB could promote breast cancer invasion and metastasis^32^. The inactivation of GSDMB could impair the aggressiveness of HER2-positive breast cancer 33. Whereas GSDMD was activated, it induced pyroptosis in TNBC and impaired the proliferation capacity^34^, ^35^. A similar effect was also observed of GSMDE on breast cancer. Enhanced generation of ROS could induce GSDME activation, and promote pyroptosis in TNBC^36^, ^37^. Breast cancer suppressor p53 could activate GSDME and inhibit CDK7 to suppress breast cancer survival 38. *In vivo*, anti-tumor immunity could be stimulated through GSDME activation 20, suggesting an advanced anti-tumor potential of GSDMD and GSDME in breast cancer.

In this study, overexpressed UCP1 causes mitochondrial swelling and membrane potential to decrease, implying mitochondrial destruction and dysfunction, which lead to the activation of GSDMs. The expression of NLRC4 and the activator of GSDMD, caspase-1^39^, did not change after UCP1 overexpression in TNBC (Fig. S3). We, therefore, assumed that GSDME, but not GSDMD, had been activated in this progress. Activated Caspase-3 and cleaved GSDME were increased after UCP1 overexpression in TNBC, suggesting that GSDME is involved in the UCP1-mediated regulation of TNBC. Cell proliferation inhibition and cell cycle arrest were reversed after impaired GSDME activation, further confirming the crucial role of GSDME instead of the mitophagy in TNBC cell proliferation.

GSDME, an inflammation-related protein possessing pore formatting capacity, induces cell swelling and fracture, showing its anti-tumor potential. Moreover, expression of GSDME was higher in lobular adenocarcinomas than ductal adenocarcinomas 40, a subtype with higher malignant than the former. Methylated GSDME could increase the risk of lymph node metastasis in breast cancer 41, indicating anti-tumor potentials of GSDME in the regulation of breast cancer. Nevertheless, GSDME downstream target in metastasis remains unclear. Further study on the regulation of GSDME in breast cancer was still in tremendous potential.

**Figure 6:**
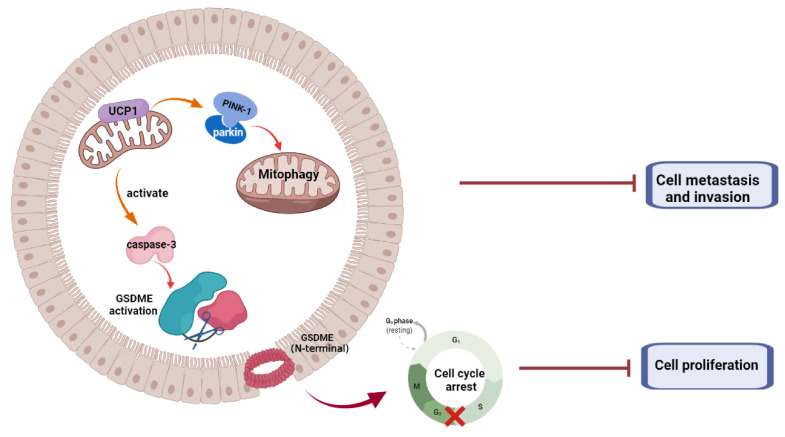
Summary of the role of UCP1 on process of TNBC

In summary, we demonstrated that UCP1 possessed an inhibitory effect on TNBC progression (Fig. 6). We found that overexpression of UCP1 induced mitochondrial destruction and dysfunction, activating mitophagy and pyroptosis, subsequently inhibiting the proliferation and metastasis of TNBC. Those results provide a Novo function and regulatory mechanism of UCP1 on TNBC and reveal the therapeutic potential of mitochondrial protein on TNBC.

## Supplementary Material

Supplementary figures.

## Figures and Tables

**Table 1:** Antibody information

Antibody name	Company	lot
UCP1	Abcam	Ab155117
HSP90	Cell Signal Technology (CST)	4877
CDK4	CST	2906
P27^kip1^	CST	3688
CyclinD1	CST	2978
MMP1	Wan lei biological technology	WL01201
VEGF-R2	Affinity	AF6281
PINK-1	CST	6946
Parkin	CST	4211
BAX	CST	2772
Bcl-2	CST	2870
Caspase-3	CST	9662
GSDME	Abcam	Ab215191
