# Supplementary material for: Mitochondrial Protein UCP1 Inhibits the Malignant Behaviors of Triple-negative Breast Cancer through Activation of Mitophagy and Pyroptosis
Source: Int J Biol Sci. 2022 Apr 18;18(7):2949–61. doi: 10.7150/ijbs.68438 (PMC9066108; doi:10.7150/ijbs.68438)
Supplement: Supplementary file 1 — Supplementary figures. [file ijbsv18p2949s1.pdf]

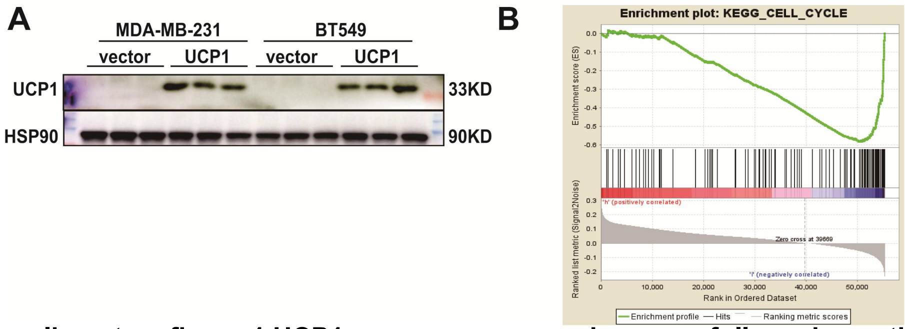

Supplimentary figure 1.UCP1 was overexpressed successfully and negative related to cell cycle pathway.(A) Protein expression of UCP1 in MDA-MB-231 and BT549 with vector or UCP1 overexpressed.**(B)**KEGG enrichment analysis of UCP1 on breast cancer in cell cycle pathway.cut off value: fold change>2 and p<0.05.

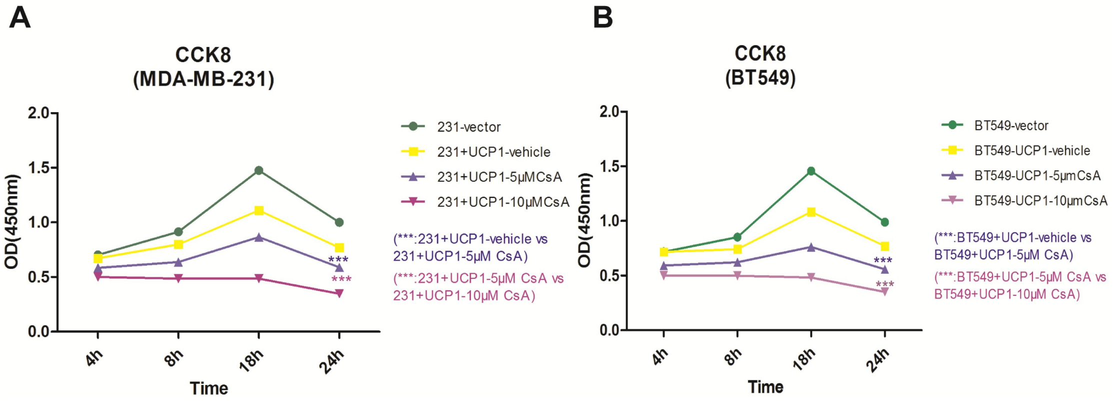

Supplimentary figure 2.Proliferation potential of TNBC cell line with UCP1 overexpressed did not change after CsA treated. (A)CCK8 assay of MDA-MB-231 and **(B)**BT549 cells with treated as descirbed.***:p<0.01

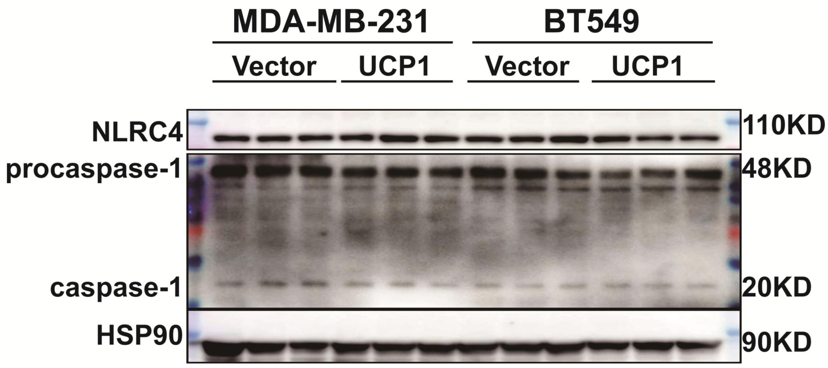

Supplimentary figure 3.NLRC4 and caspase-1 did not change after UCP1 overexpressed. Protein expression of NLRC4 and caspase-1 in MDA-MB-231 and BT549 with vector or UCP1 overexpressed.

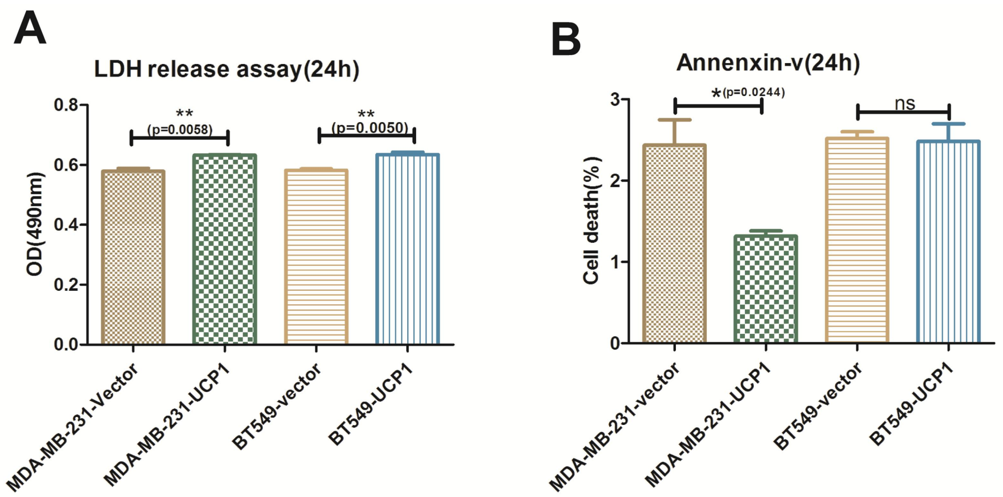

Supplimentary figure 4.Non-apoptosis pathway may actived after UCP1 overexpressed in TNBC.(A) LDH release of MDA-MB-231 and BT549 with vector or UCP1 overexpressed.**(B)** Cell apoptosis rate of MDA-MB-231 and BT549 with vector or UCP1 overexpressed.*:p<0.05,**p<0.01
